# Transcriptome Analysis Reveals the Crucial Role of Phenylalanine Ammonia-Lyase in Low Temperature Response in *Ammopiptanthus mongolicus*

**DOI:** 10.3390/genes15111465

**Published:** 2024-11-13

**Authors:** Ning Wang, Yilin Zhu, Yijun Zhou, Fei Gao, Suxia Cui

**Affiliations:** 1 College of Life Sciences, Capital Normal University, Haidian District, Beijing 100048, China; wzpn123@163.com; 2 College of Life and Environmental Sciences, Minzu University of China, Haidian District, Beijing 100081, China; 22011910@muc.edu.cn (Y.Z.); zhouyijun@muc.edu.cn (Y.Z.); 3 Beijing Key Laboratory of Plant Gene Resources and Biotechnology for Carbon Reduction and Environmental Improvement, Haidian District, Beijing 100048, China

**Keywords:** *Ammopiptanthus mongolicus*, phenylalanine ammonia-lyase, transcriptome, low temperature

## Abstract

**Background**: *Ammopiptanthus mongolicus* is a rare temperate evergreen shrub with high tolerance to low temperature, and understanding the related gene expression regulatory network can help advance research on the mechanisms of plant tolerance to abiotic stress. **Methods:** Here, time-course transcriptome analysis was applied to investigate the gene expression network in *A. mongolicus* under low temperature stress. **Results**: A total of 12,606 differentially expressed genes (DEGs) were identified at four time-points during low temperature stress treatment, and multiple pathways, such as plant hormones, secondary metabolism, and cell membranes, were significantly enriched in the DEGs. Trend analysis found that the expression level of genes in cluster 19 continued to upregulate under low temperatures, and the genes in cluster 19 were significantly enriched in plant hormone signaling and secondary metabolic pathways. Based on the transcriptome data, the expression profiles of the genes in abscisic acid, salicylic acid, and flavonoid metabolic pathways were analyzed. It was found that biosynthesis of abscisic acid and flavonoids may play crucial roles in the response to low temperature stress. Furthermore, members of the phenylalanine ammonia-lyase (PAL) family in *A. mongolicus* were systematically identified and their structures and evolution were characterized. Analysis of cis-acting elements showed that the *PAL* genes in *A. mongolicus* were closely related to abiotic stress response. Expression pattern analysis showed that *PAL* genes responded to various environmental stresses, such as low temperature, supporting their involvement in the low temperature response in *A. mongolicus*. **Conclusions**: Our study provides important data for understanding the mechanisms of tolerance to low temperatures in *A. mongolicus.*

## 1. Introduction

As stationary organisms, plants are vulnerable to numerous environmental stressors [1,2,3]. With ongoing global warming and an increase in various drastic climate alterations, the occurrence of low temperature stress is becoming increasingly frequent. Low temperature stress can affect plant growing status and regional distribution, reduce plant productivity, and thus threaten global agricultural production [4,5,6]. For instance, for each 1 °C increases in average world temperature, the total yield of rice, soybean, wheat, and corn will drop by an average of 3.2%, 3.1%, 6.0%, and 7.4%, respectively [7]. In temperate regions, low temperature conditions can lead to a decrease of about 30–40% in rice yield [8]. Low temperature stress can occur at multiple stages of plant development, hindering seed germination, damaging photosynthetic capacity, inducing incomplete development of panicles, and causing significant damage to the growth status and yield of crops [9,10].

To accommodate to rapid ambient changes, plants developed various adaptation strategies. Studies have shown that plants can regulate metabolism, alter membrane composition, activate reactive oxygen species eliminating systems, and regulate hormone related signaling pathways by changing the levels of many genes [11,12,13]. During overwintering period, temperate plants tend to synthesize more cryoprotectant molecules, like sugar alcohols, soluble sugars, and some small nitrogen-containing molecules [14]. Cold stress increases the production of phenolic substances, which are then incorporated in cell wall as suberin or lignin, increasing the cell’s tolerance to low temperatures [15]. In addition, multiple plant hormones, especially abscisic acid (ABA), brassinosteroid (BR), jasmonic acid (JA), and salicylic acid (SA), also play important roles in plants’ resistance to environmental stress [11]. Recently, research on low temperature response has found that low temperature inducible genes play crucial parts in low temperature adaptation in tomato. The transcription factor CRT/DRE binding factor (CBF) and its upstream transcription factor CBF expression inducing factor 1 (ICE1) work together to activate the transcription of low temperature modulated genes and enhance plant tolerance to low temperature conditions [16]. Presently, *ICE1* overexpressing transgenic plants, including *Arabidopsis thaliana* [17], rice [18], tobacco [19], and cucumber [20] have higher cold resistance than wild-type plants.

There is considerable variation in the tolerance of different plants to low temperature stress. Many economical plants including soybean, corn, rice, cotton, and tomato are sensitive to low temperatures [21]. Studies have shown that the production of rice is highly susceptible to temperature fluctuations, and prolonged cold stress promotes lipid peroxidation and hindered growth [22].The model plant *A. thaliana* is widely recognized as a cold tolerant species, capable of growing at low temperatures as low as 15 °C and surviving at freezing temperatures [23,24]. Some plants species have a high degree of tolerance to low temperatures, such as *Ammopiptanthus mongolicus* and *Euonymus japonicus*, which can withstand temperatures below −10 °C without shedding leaves in temperate regions during winter. It was reported that, when the lowest temperature drops to −20 °C in winter, *A. mongolicus* can grow with normal leaves and maintain photosynthesis activity [25]. Although a large amount of transcriptomic analysis has been conducted to reveal the molecular mechanism associated with the low temperature response of plants, most of these works were focused on model plants like *Arabidopsis* and rice.

*A. mongolicus*, belonging to the legume family, is one of the rare evergreen broad-leaved woody plants in temperate regions in China. It is a relict species of the Paleogene period, endemic to central Asia [26]. *A. mongolicus* has strong tolerance to drought, high and low temperature, wind stress, and sand burial, and is mainly growing in fixed sandy land, sandy and rocky slopes [27]. *A. mongolicus* is an important material for studying plant stress resistance mechanisms and identifying stress resistant genes, with significant scientific research value. In recent years, research on the physiological, biochemical, and molecular mechanisms of drought and cold tolerance in *A. mongolicus*, has received attentions of several research teams [27,28,29,30]. Multiple stress tolerance related genes such as *AmLEA* [31], *AmCBL1* [30], and *AmNHX2* [32] have been isolated and subjected to transgenic functional analysis. Especially, multi omics method was utilized to study the dynamic changes in protein and metabolite levels in apoplast of *A. mongolicus* under winter freezing stress, as well as the changes in related gene expression [33]. However, there is currently a lack of time-course gene expression analysis of the response to low temperature stress in *A. mongolicus*.

To analyze the gene expression regulation network related to low temperature stress response of *A. mongolicus*, in the present study, time–course transcriptome sequencing analysis was performed to identify differentially expressed genes (DEGs) and related metabolic pathways. Then, WGCNA and trend analyses were applied to identify gene cluster that may play key roles in the low temperature response of *A. mongolicus*. We also further analyzed the expression profiles of *PAL* family genes under environmental stress. This study provides important data for understanding the low temperature tolerance of *A. mongolicus*.

## 2. Materials and Methods

### 2.1. Plant Materials and Low Temperature Stress Treatment

The seeds of *Ammopiptanthus mongolicus* (Maxim.) Cheng f. were collected from Zhongwei city, Ningxia Hui Autonomous Region, China. The seeds were sowed in a plastic basin soaked with a mixture of vermiculite: perlite: nutrient soil (3:1:1), exposed to 16 h of light (23 °C)/8 h of darkness (18 °C), with a light intensity of 93 μmol·m^−2^·s^−1^. After germination, the seedlings were irrigated with a 1/2 concentration of Hoagland solution [34] every four days. After 8 weeks of germination, 60 seedlings with similar growth status were moved to a plant incubator (Percival, Perry, IA, USA) at 4 °C for stress treatment. Up to 8~10 fully developed leaves were collected from the top of each *A. mongolicus* seedling just before treatment (0 h) and after 6 h, 24 h, 72 h, and 7 days (7 d) of low temperature stress treatment. The samples were quick-frozen and stored at −80 °C for transcriptome sequencing and other analysis.

### 2.2. Transcriptomic Analysis of A. mongolicus Leaves

Total RNA extraction, quality control, and library construction process for transcriptome sequencing were conducted based on previously published literature [33]. Three independent biological replicates were performed for each group. The clean reads were mapped to *A. mongolicus* genome sequences (unpublished data) using HISAT2 (version 2.1.0) software [35]. Fragments Per Kilobase of exon model per Million mapped fragments (FPKM) was used to represent the expression level of each transcript. DEGs in *A. mongolicus* leaves were identified using DEseq2 software (version 1.18.1) [36], which used models based on negative binomial distributions. The method of Benjamini-Hochberg was used to adjust the *p*-values to control for false discovery rates. Genes that meet the following criteria were defined as DEGs: fold change value ≥ 2 and adjusted *p*-value ≤ 0.05, or fold change value ≤ 0.5 and adjusted *p*-value ≤ 0.05. Principal component analysis (PCA) was performed using FactoMineR (version 1.42) [37]. GO and KEGG analyses of DEGs were performed using the ClusterProfiler software (version 4.6.2) [38]. Trend analysis (*p* < 0.05 was considered statistically significant, and the number of trends was set to 20) and weighted gene co-expression network analysis (WGCNA) were implemented by the OmicShare online tool (https://www.omicshare.com/tools/, accessed on 11 August 2024).

### 2.3. Identification of PAL Family Genes in A. mongolicus

We used Hidden Markov Model (HMM) and BlastP to identify PAL family members in *A. mongolicus* genome with refer to a method described previously [39]. HMM profile of the PAL domain (PF00221: Aromatic amino acid lyase) was downloaded from the Pfam database (http://pfam.xfam.org/, accessed on 2 September 2024). The HMMER 3.0 software was used to identify the PAL family members from the genome sequence of *A. mongolicus* with a threshold of *E* < 10^−5^. Sequences of four *Arabidopsis* PAL proteins were acquired from The Arabidopsis Information Resource (TAIR) [40], and then subjected to BlastP against the *A. mongolicus* genome with a threshold of *E* < 10^−5^. Finally, PAL members in *A. mongolicus* were determined take the intersection of the two results.

### 2.4. Characteristic Analysis of PAL Members in A. mongolicus

ProtParam [41] was used to predict the theoretical pI, molecular weight, instability index, and grand average of hydropathy (GRAVY) of PAL proteins. TBtools software (version 2.127) [42] was used to draw the chromosome localization map and exon-intron structure map of *PAL* genes. Plant mPLoc [43] was used to analyze subcellular localization of PAL proteins. MEME [44] and NCBI CDD [45] were used to analyze the conserved motifs and protein domain, respectively. SOPMA [46] and SWISS-MODEL [47] were used to analyze the secondary structure and tertiary structure, respectively. To investigate the putative cis-acting elements in the *AmPAL* promoter, sequences of 2000 bp upstream of the initiation codon of the *PAL* genes in *A. mongolicus* were submitted to PlantCARE web tool [48], and the results were displayed using TBtools software (version 2.127).

### 2.5. Phylogenetic Analysis

To explore the evolutionary relationship of the PAL members, a phylogenetic tree using the PAL proteins from *A. mongolicus*, *A. thaliana*, *Glycine max*, *Oryza sativa*, and *Zea mays* were constructed. The sequence alignment was executed using Clustal W [49]. The phylogenetic tree was constructed using MEGA 11 software (version 11.0.13) [50] with the neighbor-joining method and 1000 replicate iterations. The Interactive Tree Of Life (iTOL) [51] was used to visualize the evolutionary tree [52].

### 2.6. Expression Analysis of PAL Genes in A. mongolicus Based on Released Transcriptome Data

The transcriptome data of different seasons (summer, autumn, and winter) were downloaded from NCBI SRA under nine consecutive accession numbers from SRR16479821 to SRR16479829. Transcript abundance was calculated using Kallisto quant and displayed as TPM values (Transcripts Per Kilobase of exon model per Million mapped reads).

### 2.7. Expression Analysis of PAL Genes in A. mongolicus Using qRT-PCR Analysis

The 8-week-old *A. mongolicus* seedlings were subjected to simulated drought stress with 20% PEG 6000, low temperature stress with 4 °C, high salt stress with 300 mM NaCl, and mechanical damage stress by piercing six parallel holes on both sides of the main leaf vein of *A. mongolicus* seedlings with sterile needles. One gram of leaves and one gram of root tissues were harvested at time-points 0 h, 6 h, 24 h, and 72 h after treatment, frozen in liquid nitrogen, and stored at −80 °C for total RNA extraction and qRT-PCR analysis.

RNA extraction and qRT-PCR analysis were conducted according to a method reported in previous studies [53]. Three biological replicates were set for each group, and three technical replicates were set for each sample. *AmeIF1* gene was used as the reference gene, and the relative expression level of a gene was calculated using the 2^−△△Ct^ method. Statistical analysis was performed using SPSS software (SPSS version 25; IBM, Chicago, IL, USA). *p* ≤ 0.05 was considered to be significant. The primers used in qRT-PCR analysis for PAL gene are shown in the Table S1.

## 3. Results

### 3.1. Transcriptomic Analysis of the Response to Low Temperature Stress in A. mongolicus

**Figure 1:**
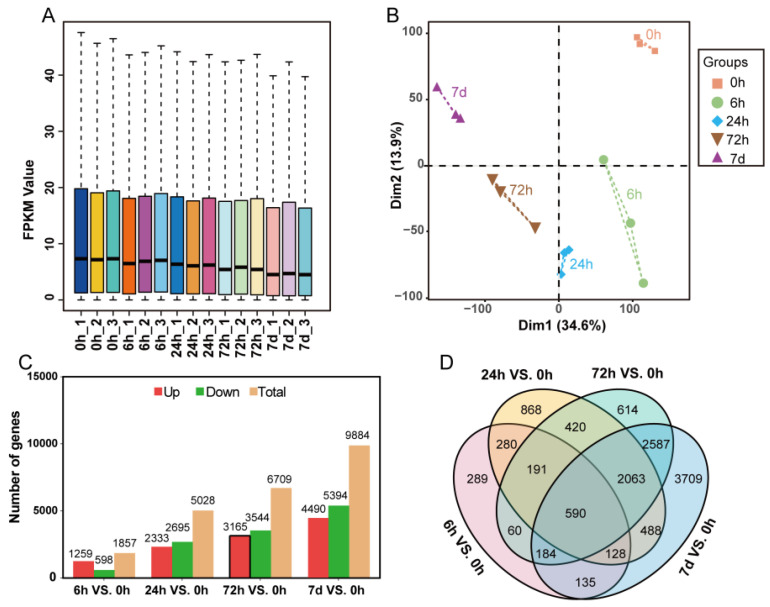
Transcriptomics analysis of *A. mongolicus* leaves under low temperature stress. (**A**) Gene expression abundance at time-points 0 h, 6 h 24 h, 72 h, and 7 days. The horizontal axis represents the sample name, while the vertical axis represents the gene expression level. The bold line represents the median of the expression levels (FPKM). (**B**) PCA analysis of the expression levels of all genes. Different colors represent different groups. Three biological replicates in the same group are connected by dashed lines. (**C**) The number of DEGs at different time-points (6 h, 24 h, 72 h, and 7 d). Red represents upregulated genes, green represents downregulated genes, and light orange represents all DEGs. (**D**) Venn diagram analysis of the DEGs identified at different time-points (6 h, 24 h, 72 h, and 7 d, represented by pink, yellow, green, and blue, respectively).

RNA-seq was used to study the transcriptome changes of *A. mongolicus* under low temperature stress. A total of 103.98 Gb clean data were obtained and the percentage of high-quality reads (Q30) exceeded 94.72%. The expression abundance of genes in all samples was shown in Figure 1A, and the distribution of gene expression levels among different groups was similar. PCA analysis indicated that the three biological replicates of each group were significantly clustered together, and significant separation occurred between different sample groups (Figure 1B). The genes with |log_2_ (FC)| ≥ 1 and adjusted *p*-value ≤ 0.05 were considered to be DEGs. A total of 12,606 DEGs were identified (at least differentially expressed in any group after low temperature stress), which might be involved in the response of *A. mongolicus* to low temperature stress. The number of DEGs gradually increased with prolonged stress time. Compared with 0 h, 1857, 5028, 6709, and 9884 DEGs were identified in the 6 h, 24 h, 72 h, and 7 d groups, respectively (Figure 1C). The Venn diagram showed that 590 DEGs were detected at all the four time-points, with 289, 868, 614, and 3709 were detected at time-points 6 h, 24 h, 72 h, and 7 d, respectively (Figure 1D).

### 3.2. GO and KEGG Enrichment Analysis of DEGs in A. mongolicus Under Low Temperature Stress

**Figure 2:**
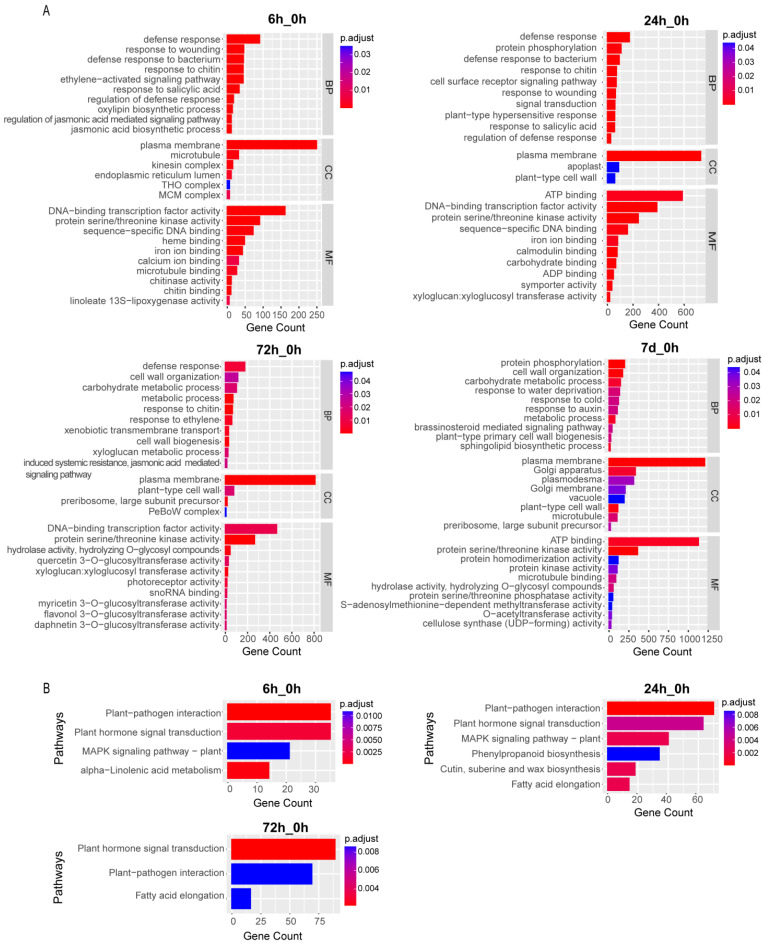
GO and KEGG enrichment analysis of the identified DEGs in *A. mongolicus* under low temperature stress. (**A**) GO enrichment analysis. Terms with an adjusted *p*-value less than 0.05 are identified as significantly enriched GO terms. The vertical axis represents the GO entry, the horizontal axis represents the number of enriched genes in that entry, color represents adjusted *p*-value, and the redder the color, the more significant it is. Only the most significant 30 GO entries are displayed (10 each for BP, CC, and MF; if less than 10, all GO entries were shown). (**B**) KEGG enrichment analysis. Pathway entries with an adjusted *p*-value less than 0.05 are identified as significantly enriched pathway. The vertical axis represents the pathway name, the horizontal axis represents the number of genes enriched in the pathway, and the color represents the adjusted *p*-value. The redder the color, the more significant the result. Only the 30 most significant pathways are displayed. If less than 30, all KEGG pathways are shown.

To identify the biological functions or pathways that are significantly enriched in DEGs identified at different time-points, GO and KEGG enrichment analyses were performed (Figure 2 and Figure S1). Compared with 0 h, a total of 77, 76, 37, and 31 GO terms were enriched in DEGs identified in 6 h, 24 h, 72 h, and 7 d groups (Figure S1). The number of enriched entries in the 6 h_0 h and 24 h_0 h comparison groups were more than twice that of the 72 h_0 h and 7 d_0 h comparison groups. In all comparison groups, the three entries of “response to water depletion”, “plasma membrane”, and “protein serine/threonine kinase activity” were significantly enriched. It was found that 41 entries were significantly enriched only in the 6 h_0 h comparison group, including “jasmonic acid biosynthetic process”, “response to brassinosteroid”, “activation of immune response”, and “response to biotic stimulus”, and 13 items were significantly enriched only in the 7 d_0 h comparison group, including “sphingolipid biosynthetic process”, “plant type primary cell wall biogenesis”, and “response to auxin”. In addition, we found that GO terms related to abscisic acid (“abscisic acid activated signaling pathway” and “response to abscisic acid”) were significantly enriched in the 24 h_0 h, 72 h_0 h, and 7 d_0 h comparison groups, indicating that the abscisic acid related signaling pathway played a part in low temperature response of *A. mongolicus*.

KEGG analysis revealed that seven pathways (Figure S1) were overrepresented in all DEGs. Compared with 0 h, 4, 6, and 3 metabolic pathways were presented in the 6 h, 24 h, and 72 h treatment groups, respectively, while no enriched metabolic pathway was detected in 7 d treatment group. Among the overrepresented metabolic pathways, the “plant-pathogen interaction” and “plant hormone signal transduction” pathways were significantly overrepresented in the 6 h_0 h, 24 h_0 h, and 72 h_0 h comparison groups. “Alpha-Linolenic acid metabolism” was specifically overrepresented in the 6 h_0 h group. “Cutin, suberine, and wax biosynthesis” and “Phenylpropanoid biosynthesis” were specifically overrepresented in the 24 h_0 h group.

### 3.3. WGCNA Analysis of Low Temperature Response in A. mongolicus

**Figure 3:**
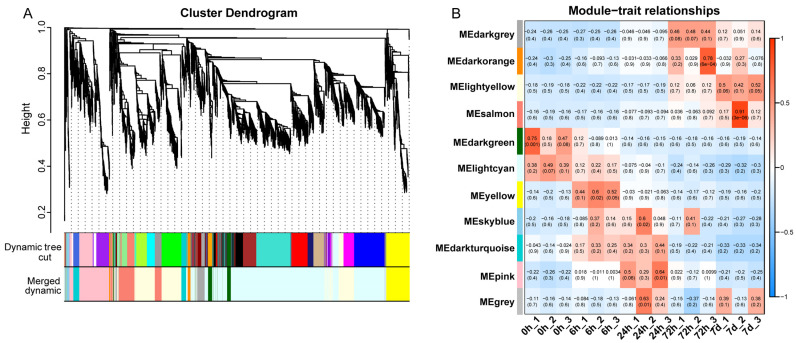
WGCNA analysis of all identified DEGs in leaves of *A. mongolicus* under low temperature stress. (**A**) Clustering and module delineation. (**B**) Module–time point relationship diagram. Each row corresponds to a module, and each column represents a time–point. The correlation coefficients (*r*-values) were shown above, and the *p*-values were shown below. Red represents positive correlation, and blue represents negative correlation.

To understand the transcriptional regulatory network of *A. mongolicus* in response to low temperature stress, WGCNA analysis was performed on DEGs in all samples. The cut-off of R^2^ was set to be 0.85 and soft-thresholding power (β) was set at 15. All DEGs were divided into 11 modules represented by different colors (Figure 3A). The number of genes in each module varies greatly, with the two modules with the highest number being the lightcyan module (1822 DEGs) and the lightyellow module (698 DEGs). To characterize the key modules related to the low temperature response of *A. mongolicus*, the correlation between these modules and temporal traits was subsequently analyzed (Figure 3B). The results showed that genes in the four modules of darkgrey, darkorange, lightyellow, and salmon exhibited similar expression patterns in response to low temperature stress in *A. mongolicus*. They were negatively associated with 0 h, 6 h, and 24 h, and positively correlated with 72 h and 7 d. The darkgreen and lightcyan modules were positively associated with 0 h and 6 h, and negatively correlated with 24 h, 72 h, and 7 d. The yellow module was positively correlated with 6 h and negatively correlated with other time-points. The skyblue and darkturquoise modules were positively associated with 6 h and 24 h, and negatively associated with other time-points. It is worth noting that the lightcyan module was positively correlated with 0 h (*r* = 0.42) and 6 h (*r* = 0.17), and negatively correlated with 24 h (*r* = −0.07), 72 h (*r* = −0.21), and 7 d (*r* = −0.30), and the correlation coefficient gradually decreased. The lightyellow module was positively correlated with 72 h (*r* = 0.10) and 7 d (*r* = 0.48), and negatively correlated with 0 h (*r* = −0.18), 6 h (*r* = −0.22), and 24 h (*r* = −0.18). The above data suggested that genes in the lightcyan and lightyellow modules were closely associated with the response of *A. mongolicus* to low temperature stress.

To investigate the potential function of genes of two key modules (lightyellow and lightcyan) during low temperature stress, we conducted GO and KEGG analyses (Figure S2). The genes in the lightyellow module were annotated to 46 GO terms, including 21 biological process terms, 13 cellular component terms, and 12 molecular function terms. The three most enriched terms for biological process were “response to abiotic stimulus”, “lipid biosynthesis process”, and “response to light stimulus”; For the cellular component, “nucleus”, “plastid”, and “chloroplast” were the top three enriched entries; For molecular function, the top three enriched entries were “transition metal ion binding”, “transporter activity”, and “transmembrane transporter activity”. A total of 136 metabolic pathways were annotated for all DEGs through KEGG analysis, with “MAPK signalling pathway - plant”, “circadian rhythm—plant”, “plant hormone signal transduction”, “Glycerophospholipid metabolism” and “biosynthesis of secondary metabolites” being the top five significantly enriched pathways. The genes in the lightcyan module were annotated with a total of 48 GO terms, including 19 biological process terms, 17 cellular component terms, and 12 molecular function terms. For biological process, the three most enriched terms were “oxidation-reduction process”, “response to hormone”, and “cell response to chemical stimulus”; for the cellular component, “cell periphery”, “cell wall”, and “external encapsulating structure” were the top three enriched entries; for molecular function, the top three enriched terms were “oxidoreductase activity”, “transferase activity, transferring acyl groups”, and “tetrapyrrole binding”. KEGG enrichment analysis showed that a total of 109 metabolic pathways were annotated, with “fatty acid elongation”, “fatty acid metabolism”, “metabolic pathways”, “photosynthesis - antenna proteins”, and “biosynthesis of secondary metabolites” being the top five significantly enriched metabolic pathways.

### 3.4. Trend Analysis of Genes in A. mongolicus Under Low Temperature Stress

**Figure 4:**
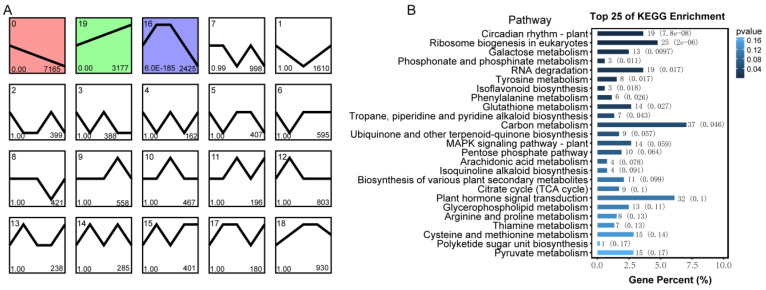
Trend analysis of genes in *A. mongolicus* under low temperature stress. (**A**) The significance and gene number of each module. Profiles ordered based on the *p*-value significance of number of genes assigned versus expected. The number in the bottom left corner of each box represents the *p*-value, and the number in the bottom right corner represents the number of genes. The first three colored boxes are those with *p*-values less than 0.05. (**B**) KEGG enrichment analysis of genes included in module 19. *p*-value < 0.05 indicated a significantly enriched pathway. The number after the bar represents the number of enriched genes, and the number in parentheses represents the *p*-value.

To investigate the gene expression trends upon low temperature stress, we conducted trend analysis on all expressed genes in *A. mongolicus*. All genes were aggregated into 20 modules (Figure 4A). Among them, the trends of the genes enriched in modules 0, 19 and 16 were statistically significant. The genes in module 0 are continuously downregulated in expression with prolonged treatment time. The genes enriched in module 19, which contains 3177 genes showing an upward trend, suggest that the genes in this module were involved in the response of *A. mongolicus* to low temperature stress. In addition, the genes in module 16 were upregulated in the early stages of low temperature stress, and then downregulated in the late stages of stress, with expression levels lower than time-point 0 h.

To determine the metabolic pathways represented by genes with monotonically upregulated and downregulated expression, we conducted GO and KEGG functional enrichment analysis on the genes in module 0 and 19 (Figure 4B, Figures S3 and S4). Based on the results in Figure 4B, we found that the genes in module 19 were significantly enriched in 11 pathways. The top three most significantly enriched pathways were “Circadian rhythm—plant”, “Ribosome biogenesis in eukaryotes”, and “Galactose metabolism”. In addition, multiple pathways related to secondary metabolism, such as “Isoflavonoid biosynthesis” and “Phenylalanine metabolism”, “Tropane, piperidine and pyridine alkaloid biosynthesis”, were also significantly enriched.

### 3.5. Expression Profiles of the Genes Involved in Biosynthesis and Signaling of ABA and SA in A. mongolicus

**Figure 5:**
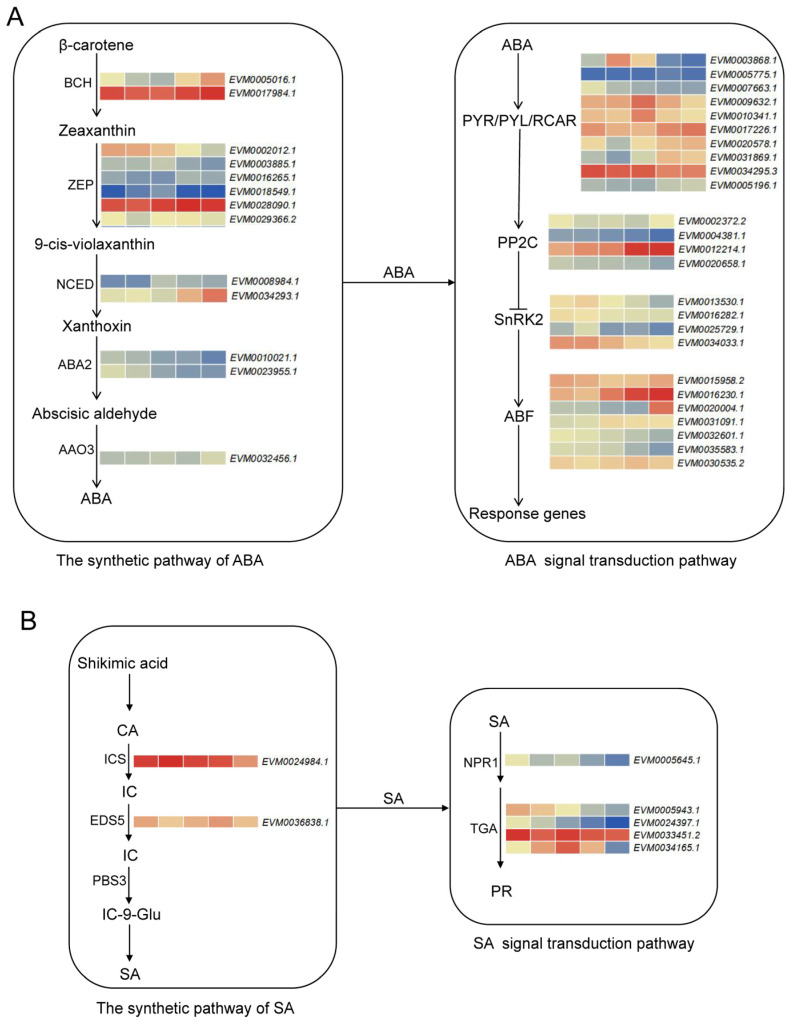
The alterations in the transcription levels of genes related to ABA and SA synthesis pathway and signaling pathway in leaves of *A. mongolicus* after low temperature treatment. The expression level of genes (FPKM value) is represented by a heatmap, where red represents high expression and blue represents low expression. (**A**) ABA biosynthesis (left) and signaling pathway (right). (**B**) The biosynthesis of SA (left) and signaling pathway (right). Abbreviations: β-carotene hydroxylase (BCH), zeaxanthin epoxidase (ZEP), 9-cis-epoxycarotenoid dioxygenase (NCED), xanthoxin dehydrogenase (ABA2), abscisic-aldehyde oxidase 3 (AAO3), abscisic acid receptor PYR/PYL family (PYR/PYL/RCAR), protein phosphatase 2C (PP2C), Serine/threonine-protein kinase (SnRK2), ABA-responsive element binding factors (ABF), isochorismate synthase (ICS), enhanced disease susceptibility 5 (EDS5), avrPphB susceptible 3 (PBS3), regulatory protein NPR1 (NPR1), TGA transcription factor TGA (TGA), chorismate acid (CA), isochorismate (IC), and isochorismoyl-9-glutamate (IC-9-Glu).

Analysis of DEGs revealed that the signaling pathways of plant hormones such as abscisic acid (ABA) and salicylic acid (SA) may play a part in the response of *A. mongolicus* to low temperature stress. Therefore, we conducted a detailed analysis of the alterations of the genes involved in the synthesis and signaling of these hormones (Figure 5). With the prolongation of low temperature stress time, the expression levels of *β-carotene hydroxylase* (*BCH*) and *9-cis-epoxycarotenoids dioxygenase* (*NCED*) in the ABA synthesis pathway were remarkably increased, while *xanthoxin dehydrogenase* (*ABA2*) and some *zeaxanthin epoxidase* (*ZEP*) transcripts showed a downward trend. In the process of ABA signal transduction, the changes in expression levels of different PYR/PYL/RCAR receptor transcripts were inconsistent. The expression level of *protein phosphatase 2C* (*PP2C*) gene *EVM0012214.1* was remarkably increased upon low temperature, while that of *Serine/threonine-protein kinase 2* (*SnRK2*) was generally downregulated. The genes *EVM0016230.1*, *EVM0020004.1*, and *EVM0031091.1* encoding ABF transcription factors were remarkably increased upon low temperature treatment.

In the SA synthesis pathway, *isocitrate synthase* (*ICS*) was continuously downregulated under low temperature stress, while *enhanced disease susceptibility 5* (*EDS5)* showed no significant change. In the SA signal transduction pathway, the expression levels of SA receptor *NPR1* (*regulatory protein NPR1*) and *transcription factor TGA* were also consistently downregulated. These results may be related to the decreased disease resistance of *A. mongolicus* under low temperature stress.

### 3.6. Expression Profiles of Transcripts Involved in Flavonoids Synthesis in A. mongolicus

**Figure 6:**
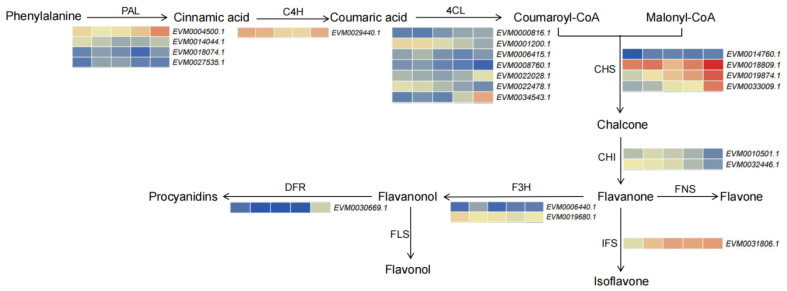
Alterations of expression levels of genes associated with flavonoid pathway in leaves of *A. mongolicus* under low temperature stress. The expression level of genes (FPKM value) is represented by a heatmap, where red represents high expression and blue represents low expression. Abbreviations: dihydroflavonol 4-reductase (DFR), flavone synthase (FNS), chalcone synthase (CHS), phenylalanine ammonia-lyase (PAL), 4-coumarate-CoA ligase (4CL), chalcone isomerase (CHI), flavanone 3-hydroxylase (F3H), flavonol synthase (FLS), cinnamate 4-hydroxylase (C4H), and isoflavone synthase (IFS).

Flavonoids are a type of secondary metabolite widely distributed in plants. They have low molecular weight and have been shown to participate in plant stress response to various stressors, such as low temperature, drought, freezing, and ultraviolet radiation [54]. Here, we found that the terms related to secondary metabolism and flavonoid biosynthesis were also significantly enriched in *A. mongolicus* under low temperature stress. Therefore, we conducted a detailed analysis of the transcription of genes associated with flavonoid biosynthesis (Figure 6). It was found that the transcription levels of *PAL* (*EVM0004500.1*), *4CL* (*EVM000816.1*, *EVM0022028.1*, *EVM0034543.1*), *CHS* (*EVM0018809.1*, *EVM0019874.1*, and *EVM0033009.1*), *DFR* (*EVM0030669.1*), and *IFS* (*EVM0031806.1*) showed a fluctuating upward trend with the prolongation of low temperature stress time. Compared with the unstressed treatment, they were significantly upregulated after 7 d of low temperature stress. The expression level of *C4H* (*EVM0029440.1*) was continuously downregulated before time-point 24 h, and returned to the level comparable to time-point 0 h.

### 3.7. Identification of PAL Family Genes in A. mongolicus

**Figure 7:**
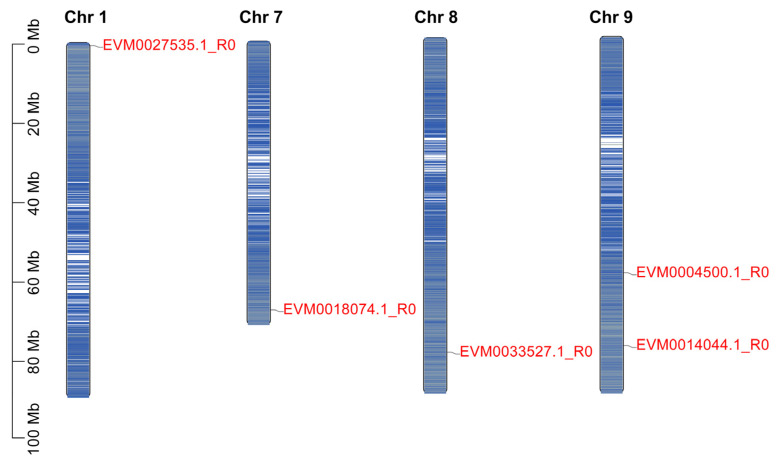
Distribution of *PAL* genes on chromosomes of *A. mongolicus*. IDs of *PAL* genes are displayed on the right side of chromosomes (marked in red font). The genes on chromosomes are represented by blue bars, with darker blue indicating higher gene density at this location.

Considering that phenylalanine ammonia-lyase (PAL) is one of the important rate-limiting enzymes in the phenylpropanoid pathway of plants, we further conducted the systematic identification of *PAL* genes in *A. mongolicus*. A total of five *PAL* genes were identified from *A. mongolicus* genome, namely *EVM0027535.1_R0*, *EVM0018074.1_R0*, *EVM0033527.1_R0*, *EVM0004500.1_R0* and *EVM0014044.1_R0* (Table 1). According to the physical location of *AmPAL* members on chromosomes, they were named *AmPAL1*, *AmPAL2*, *AmPAL3*, *AmPAL4*, and *AmPAL5* (Figure 7). Chromosome mapping showed that all *AmPAL* members were unevenly distributed on four chromosomes. There was one *AmPAL* gene on chromosomes 1, 7, and 8 respectively, while there were two *AmPAL* genes on chromosome 9. The amino acid sequence length of PAL family members ranged from 522 to 724 Aa, and the molecular weights of the proteins ranged from 56.32 to 78.85 kD. All the PAL proteins had isoelectric points less than 7.0, indicating they were all acidic proteins. Hydrophilicity analysis showed that the GRAVY values of all PAL proteins were negative, indicating that they were all hydrophilic proteins. The subcellular localization prediction results showed that all PAL proteins were located in the cytoplasm, which was consistent with the subcellular localization results of PAL proteins in other species [55,56,57].

**Figure 8:**
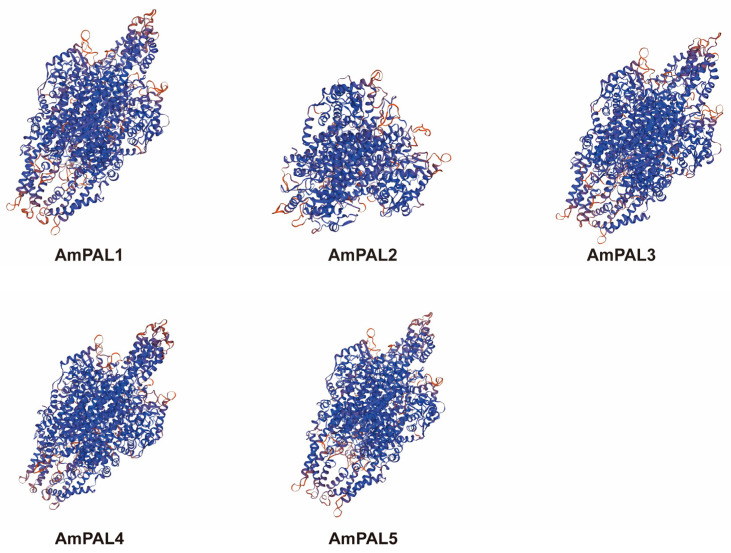
The tertiary structure of PAL proteins in *A. mongolicus*. Colors represent the confidence of structural predictions, with blue indicating high confidence and red indicating low confidence.

Protein secondary structure predictions revealed that all AmPAL proteins comprised three kinds of secondary structures: α-helix, extended strand and random coil (Table 2). AmPAL1 also contained a low proportion of β-turn. Among all the structures, the proportion of α-helix was the highest, ranging from 47.51 to 55.12%, followed by random coil and extended strand. Furthermore, the tertiary protein structures of AmPAL proteins were predicted using SWISS-MODEL (Figure 8), and the result highlighted that AmPAL1, AmPAL3, AmPAL4, and AmPAL5 shared high similarity. The similarity between AmPAL2 and other proteins was relatively low.

### 3.8. Analysis of Conserved Protein Motifs, Conserved Protein Domains and Gene Structures of AmPAL Members

**Figure 9:**
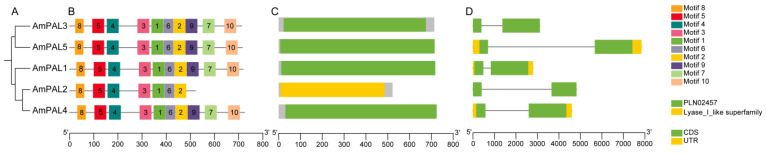
Protein and gene structures of *AmPAL* family members. (**A**) A phylogenetic tree was constructed for five *AmPAL* genes. (**B**) Conserved protein motifs of the five AmPAL proteins. Different conserved motifs with numbers 1–10 displayed in different colors. The number represents the quantity of a certain motif. (**C**) Analysis of conserved domain of AmPAL proteins. The green box represents conserved domain “PLN02457”, and the yellow box represents conserved domain “Lyase_I_like superfamily”. (**D**) Gene structure of the five *AmPAL* genes. The green box represents “coding sequence (CDS)”, and the yellow box represents “untranslated region (UTR)”.

To investigate the structure feature of *A. mongolicus* PAL proteins, we analyzed the conservative protein motifs, conserved protein domains, and gene structures of five AmPAL proteins (Figure 9). Based on the phylogenetic tree, the five AmPAL proteins were categorized into two subfamilies, and ten conserved motifs, designated as motif 1 to 10, were determined. According to the results, we found that AmPAL1, AmPAL3, AmPAL4, and AmPAL5 all contained motif 1 to motif 10, while AmPAL2 lacked motifs 7, 9, and 10. The conserved domain analysis results showed that AmPAL1, AmPAL3, AmPAL4, and AmPAL5 all contained the “PLN02457, phenylalanine ammonia lyase” domain, while AmPAL2 contained the “cl00013, Lyase_Sileke Superfamily”. It is worth noting that the “PLN02457, phenylalanine ammonia lyase “domain belongs to the “cl00013, Lyase_Sileke Superfamily”. Analysis of gene structure revealed that all five *AmPAL* genes harbored two exons and one intron.

### 3.9. Prediction and Analysis of the Cis-Acting Elements of PAL Genes in A. mongolicus

**Figure 10:**
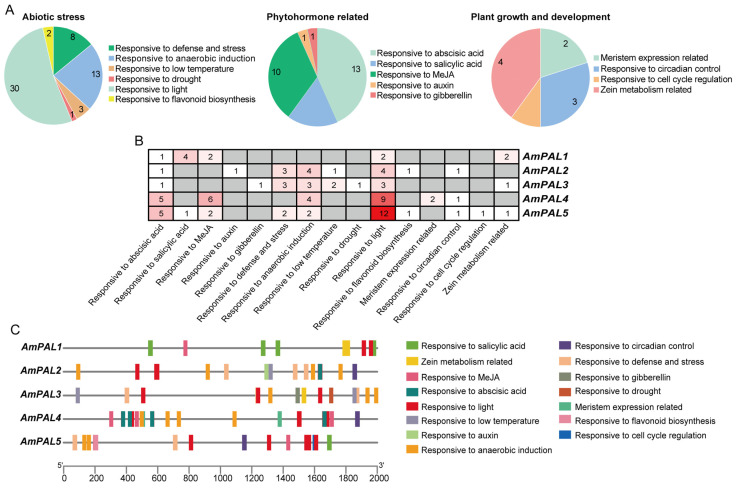
Cis-acting elements predicted in the promoters of *A. mongolicus PAL* genes. (**A**) The statistics of cis-acting elements related to abiotic stress response, plant hormones, and plant growth and development in five *PAL* genes are shown in the pie chart. (**B**) Statistics of various cis-acting elements in five *PAL* genes. The numbers in the box represent the number of cis-acting elements; (**C**) visual representation of the distribution of cis-acting elements in the five *PAL* genes. Different colors represent different cis-acting elements.

At the transcriptional level, genes are usually regulated through the interaction of cis-acting elements and trans-acting factors to ensure proper expression. In order to explore the potential biological processes associated with the *PAL* genes of *A. mongolicus*, we extracted 2000 bp sequences upstream of the start codon of *AmPAL* genes and uploaded them to PlantCARE web tool for cis-acting element prediction (Figure 10). Up to 98 cis-acting elements were predicted from the five *PAL* genes, which could be categorized into three types according to their putative roles: abiotic stress response, plant hormones, and plant growth and development (Figure 10A). Among them, the quantity of abiotic stress response elements was highest, with a number of 57. The number of plant-hormone and growth-and-development related elements was 30 and 10, respectively. The three most abundant cis-acting elements in response to abiotic stress were “responsive to light”, “responsive to anaerobic induction”, and “responsive to defense and stress”, while in plant hormone related elements were “responsive to abscisic acid”, “responsive to MeJA”, and “responsive to salicylic acid”, and in plant growth and development were “zein metabolism related”, “responsive to circadian control”, and “meristem expression related”. All five *AmPAL* genes contained cis-acting elements “responsive to abscisic acid” and “responsive to light” (Figure 10B,C).

### 3.10. Multiple Sequence Alignment and Phylogenetic Analysis of PAL Proteins in A. mongolicus

**Figure 11:**
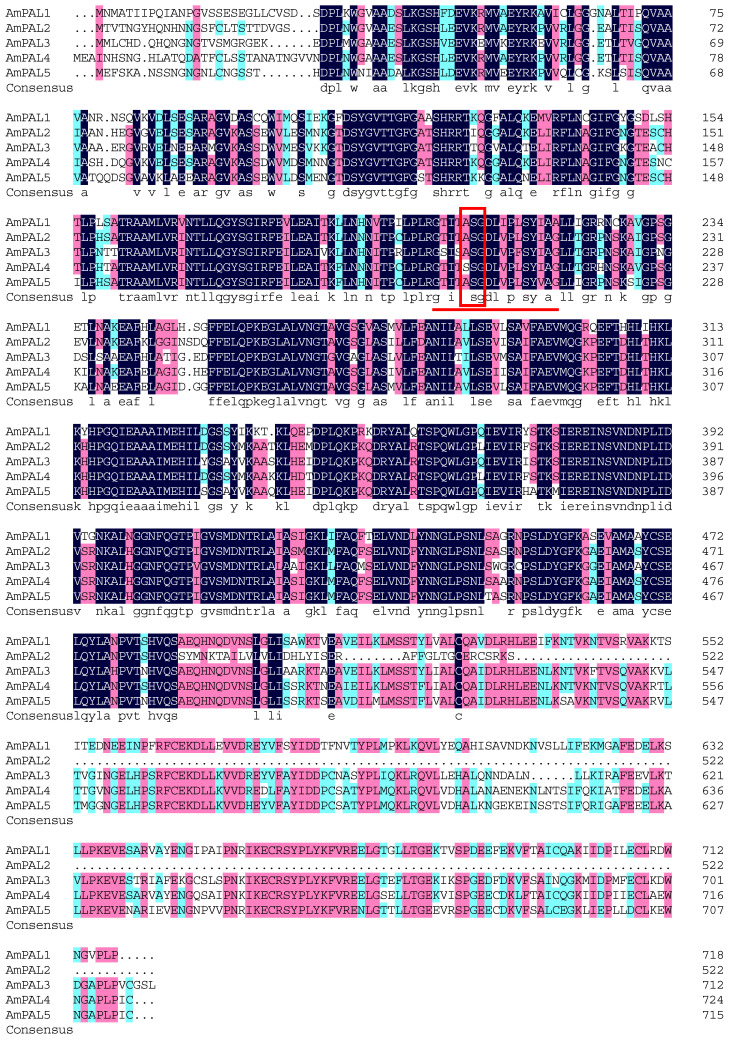
Multiple alignment of the amino acid sequences of AmPAL proteins. The underline represents the active site of PAL. The box shows the electrophilic group composed of Ala-Ser-Gly amino acid residues in PAL proteins. Black represents five identical amino acids, red represents four identical amino acids, and green represents three identical amino acids.

To detect the sequence conservation among the PAL members in *A. mongolicus*, the amino acid of five AmPAL proteins were aligned. The results showed that the amino acid sequences of the five PAL proteins were highly similar (Figure 11). Except for AmPAL4, the amino acid sequences of other AmPAL proteins contained highly conserved methylene imidazolone (MIO) electrophilic groups composed of Ala-Ser-Gly in the core domain. The results above indicate that PAL is relatively conservative in evolution.

**Figure 12:**
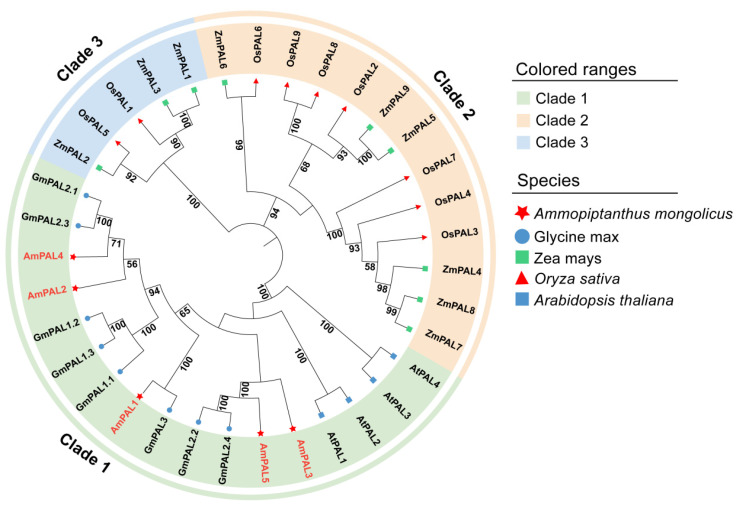
Phylogenetic tree of the PAL family members in *A. mongolicus*, *A. thaliana*, *G. max*, *O. sativa*, and *Z. mays*. The tree was generated through the MEGA 11 software by the maximum likelihood method, with 1000 bootstrap replicates. The three major phylogenetic clades were labelled by different colored backgrounds. Five species were identified with different symbols.

In order to further explore the evolutionary relationship of *PAL* genes in *A. mongolicus* and in different plant species, the maximum likelihood method was used to construct a phylogenetic tree by combining the protein sequences of five PAL proteins from *A. mongolicus* and PAL proteins from *A. thaliana*, *G. max*, *O. sativa*, and *Z. mays* (Figure 12, Table S2). The results showed that the PAL proteins of the five plants species were divided into three clusters. PAL proteins from *A. mongolicus*, *A*. *thaliana*, and *G. max*, three dicotyledonous plants, clustered together, while PAL proteins from *O. sativa*, and *Z. mays*, two monocotyledonous plants, clustered together in the other two clusters. The five PAL proteins of *A. mongolicus* were most closely related to *G. max* PAL proteins as they all belong to the legume family, indicating that they may have similar biological functions.

### 3.11. Expression Pattern Analysis of PAL Genes in A. mongolicus at Different Tissues and Under Different Stresses

**Figure 13:**
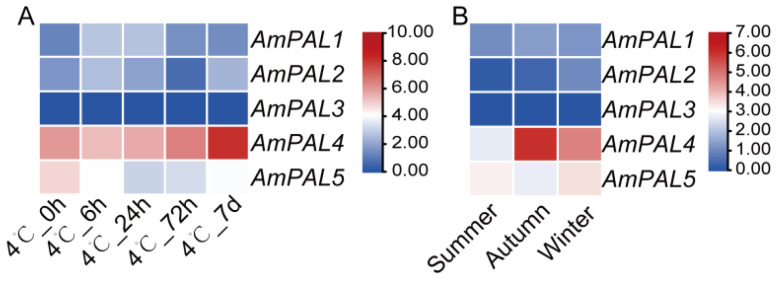
Expression pattern of *PAL* genes in *A. mongolicus* based on RNA Seq data. (**A**) The expression level changes of *PAL* genes under low temperature stress; (**B**) The expression level changes of *PAL* genes in summer, autumn, and winter. The color scale represents log_2_ transformed counts normalized by TPM, where blue indicates low expression, and red indicates high expression.

To understand the important role of *PAL* genes in response to environment stress in *A. mongolicus*, we analyzed the expression patterns of five *AmPAL* genes based on the transcriptome data obtained in this study and previous studies (Figure 13). The results indicated that the expression levels of *AmPAL2* and *AmPAL3* have always been relatively low under normal and different stress conditions, indicating that *AmPAL1*, *AmPAL4*, and *AmPAL5* are the dominant members in *PAL* family of *A. mongolicus*, especially *AmPAL4*. Under low temperature stress, the expression level of *AmPAL4* sharply increased with the prolongation of treatment time, reaching its highest expression level at 7 d, which was 4.5 times that of 0 h (Figure 13A). *AmPAL1* and *AmPAL2* first increased and then decreased. However, *AmPAL5* continued to decline, with its expression level at 7 d being only about half of that at 0 h. Based on the transcriptome data of summer, autumn, and winter, we found that only *AmPAL4* showed the most significant changes (Figure 13B). Compared with summer, the decrease in temperature in autumn led to a significant increase in *AmPAL4* expression level. In winter, the expression level of *AmPAL4* decreased, but it was still significantly higher than that in summer. The results above indicate that *PAL* genes can respond to temperature changes, with *AmPAL4* being the most responsive gene.

**Figure 14:**
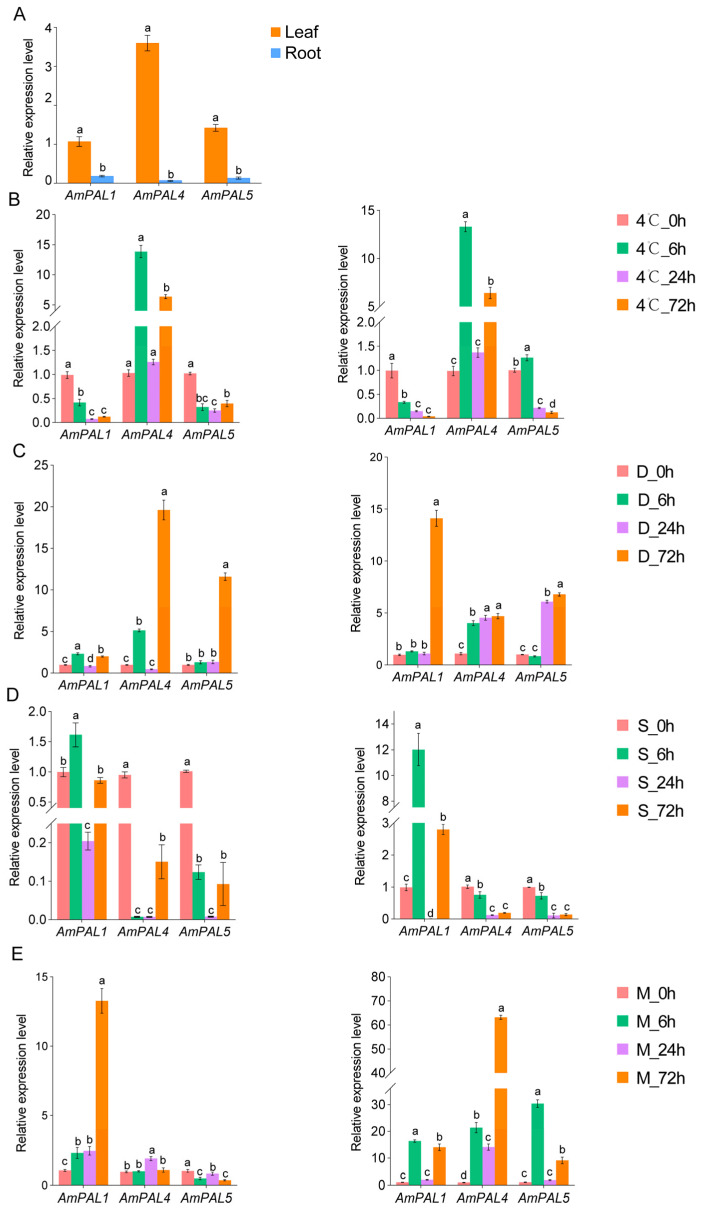
Expression pattern of *PAL* genes in *A. mongolicus* based on qRT-PCR analysis. The expression level of *PAL* genes in leaves and roots are shown in (**A**), and the expression level of *PAL* genes in *A. mongolicus* under low temperature, drought stress, salt stress, and mechanical damage stress are shown in (**B**–**E**), respectively. The pictures on the left represent the expression in leaves, and the pictures on the right represent the expression in roots. The letters on the bar chart represent statistical significance. Different letters represent significant differences between the two groups (*p* < 0.05). Abbreviations are as follows: D, drought stress; S, high salt stress; M, mechanical damage stress.

Based on the expression analysis results from RNA-seq, *AmPAL1*, *AmPAL4*, and *AmPAL5* were selected to reveal the spatiotemporal expression pattern of *PAL* genes in *A. mongolicus* through qRT-PCR analysis (Figure 14). SPSS software (SPSS version 25; IBM, Chicago, IL, USA) was used to performed statistical analysis, with *p* ≤ 0.05 was considered to be significant. We found that *AmPAL* genes were expressed in both leaves and roots, but their expression levels in leaves were much higher than in roots. Under low temperature, the expression of *AmPAL4* showed a significant response to stress in the leaves, reaching its highest level at 6 h of treatment, which was 13.46 times that of 0 h, while the expression of *AmPAL5* was continuously downregulated. In the root, both *AmPAL4*, and *AmPAL5* showed an upregulated and then downregulated expression pattern, reaching their highest values at 6 h. *AmPAL4* showed the most significant response to low temperature stress in both leaves and roots. This result was also basically consistent with the transcriptome data under low temperature stress, proving that our transcriptome data is reliable. Under simulated drought stress, the expression levels of *AmPAL1*, *AmPAL4*, and *AmPAL5* in leaves and roots were all upregulated. Under salt stress, the expression levels of *AmPAL1* and *AmPAL5* in leaves first increased and then decreased, while *AmPAL4* showed a downward trend before time-point 24 h. In the roots, *AmPAL1* showed strong response, with the expression levels reaching peak at time-point 6 h. Under mechanical damage conditions, *AmPAL1* showed the most significant response in leaves, with its expression level reaching its peak at 72 h, and the expression patterns of *AmPAL1*, *AmPAL4*, and *AmPAL5* in the roots all showed oscillatory upward trend.

## 4. Discussion

*A. mongolicus* is a unique evergreen shrub in central Asia with strong tolerance to low temperature and water deficit stress. Abiotic stress can cause drastic physiological and molecular changes in plants, including transcriptome and metabolic regulation [6]. This study investigated the gene expression changes of *A. mongolicus* upon low temperature stress at transcriptome level by setting different durations of treatment, and then focused on analyzing the expression of PAL family, a key enzyme family for flavonoid synthesis.

Transcriptome analysis can effectively reveal changes in molecular level in plants under certain environments. Significant differences were detected in abundance of a great deal of transcripts in *A. mongolicus* under low temperature stress. By using GO and KEGG functional enrichment and WGCNA analyses, it was revealed that genes responding to low temperature stress were widely involved in several biological process including secondary metabolism, hormone signaling, and flavonoid metabolism, and similar results have also been reported in low-temperature transcriptome analysis studies in other plant species [58,59,60]. GO enrichment revealed that, for cellular component, all four comparison groups were enriched to the “plasma membrane” term, which is consistent with the opinion that plant membrane system was one the most affected cellular structure. Cell membrane is involved in material transport between cells and the apoplast, and its stability under low temperature is crucial for plant cold tolerance [61]. To survive under low temperature condition, cell membranes must keep proper fluidness in a constantly changing environment. In addition, trend analysis on all expressed genes found that the transcription of the 19th cluster transcripts continued to increase with the prolongation of low temperature stress time. Functional enrichment analysis revealed that the transcripts in this cluster were also closely associated with plant hormone related signaling pathways and secondary metabolism. Taken together, our result indicated that under low temperature conditions, *A. mongolicus* could adapt low temperature stress by regulating multiple plant hormone synthesis and signaling pathways, as well as secondary metabolism including flavonoid biosynthesis.

Plant hormones are essential signaling substances that modulate plant growth and environmental stress responses [11,62,63]. Plants generate ABA with the carotenoid pathway, which is also called the “indirect pathway” [64]. In this study, with the prolongation of low temperature stress time, the transcription levels of *BCH* and *NCED* in the ABA biosynthesis pathway were greatly upregulated, indicating ABA biosynthesis was enhanced by low temperature treatment. Plants often accumulate ABA under cold stress, and the ABA level in cold tolerant plants is higher than that in cold sensitive plants [65,66,67]. Exogenous application of ABA enhances plant resistance. One research reported that exogenous abscisic acid enhanced the cold adaptation responses of grapevine [68]. BCH and NCED are important regulatory enzymes in the ABA synthesis pathway, involved in regulating ABA synthesis to enhance plant tolerance to various abiotic stresses such as drought, low temperature, and heat stress [69,70,71]. One research about *F. daltoniana* and *F. vesca* reported that ABA level was enhanced by upregulating the expression of *NCED* and downregulating the expression of *CYP707A* under low temperature condition [72]. Another report about *Arabidopsis* indicated that overexpression of *BCH* gene could improve tolerance to drought [73]. Unlike the ABA synthesis pathway, the gene expression levels of components in the ABA signaling pathway exhibited complicated patterns under low temperature stress. Among them, some transcripts of ABA receptor *PYR/PYL/RCAR* (*EVM0003868.1*, *EVM0009632.1* and *EVM0010341.1*) showed an expression pattern of first increasing and then decreasing. One dominant *PP2C* transcript gradually increased in expression level, while the expression of *SnRK2* generally showed a decreasing trend. While most transcripts of *ABF*, including *EVM0016230.1*, *EVM0020004.1*, and *EVM0031091.1*, showed a continuous elevation in transcription levels. A study on *A. mongolicus* showed that the gene encoding the ABA signaling cascade negative regulatory factor *PP2Cs* was significantly induced under water deficient conditions, which is similar to *PP2C* in this investigation [74]. Another study indicated that overexpression of *ZmPP2C2* in tobacco increased germination rate and speed, improve low-temperature tolerance, indicating that *ZmPP2C2* was a positive regulator for plant low temperature resistance [75]. In brief, our findings suggested that ABA may accumulate after low temperature stress and cause upregulation of some transcription factors downstream of the ABA signaling, thereby activating downstream responsive gene expression.

Salicylic acid (SA) is another important plant hormone involved in regulating plant growth and defense responses. The role of SA in plant-pathogen interactions has attracted extensive attention. In addition to defensive responses, SA is also important in responding to abiotic stimulus [76,77,78]. SA and its derivatives are synthesized from chorismate (derived from shikimate pathway) [77]. In this study, both the biosynthesis and signaling pathways of SA were inhibited, indicating that the pathogen resistance of *A. mongolicus* may have decreased under low temperature stress. This strategy may help *A. mongolicus* better balance growth and development under low temperature stress. A study suggested that low levels of salicylic acid can improve pollen development in tomatoes under long-term mild high temperature conditions [79]. Some plants have also experienced different situations. For example, in *Flammulina filiform*, low temperature activated the expression of SA signaling molecules (including *NPRs* and *TGAs*) and promoted the formation of fruiting bodies [80]. This indicated that the synthesis and signaling pathways of SA in plant species in response to low temperature stress depend on the genetic background of the species.

Flavonoids are a class of secondary metabolites widely present in plants, mainly including anthocyanins, flavonols, flavanols, and proanthocyanidins (PAs) or condensed tannins [81]. These compounds have a variety of functions such as antioxidant activity and plant pathogen defense [82]. Flavonoids are generated from phenylalanine through the phenylpropanoid pathway, while phenylalanine is synthesized via the shikimate pathway [83]. In this study, we found that, under low temperature stress, some key genes related to flavonoids synthesis in *A. mongolicus*, such as *PAL*, *4CL*, *CHS*, *DFR*, showed a fluctuating upward trend with the prolongation of low temperature stress time. Researches had shown that under low temperature stress, plants accumulated flavonoid metabolites through multiple pathways (such as affecting plant hormones, regulating key enzyme activity, epigenetics) to enhance their stress tolerance [84,85]. In the present study, we found that the flavonoid metabolic pathway was activated under low temperature stress, thereby contributing to the tolerance of *A. mongolicus* to low temperature.

PAL is an enzyme that connects primary metabolism and phenylpropane metabolism in organisms. It is a key enzyme in phenylpropane metabolism and has been widely studied [86,87,88]. PAL catalyzes the deamination of L-phenylalanine, and the generated trans-cinnamic acid will enter the phenylpropane metabolic pathway [54]. Phenylpropane metabolism can generate various secondary metabolites such as flavonoids and lignin, and these secondary metabolites have been shown to play important roles in plant growth and development, as well as in disease and stress resistance reactions.

Our transcriptome data revealed considerable alterations in levels of multiple phenylpropanoid pathway related enzyme genes, including *PAL*, under low temperature stress. Therefore, we further conducted systematic identification, structural analysis, evolutionary analysis, and expression analysis of the *PAL* family genes in *A. mongolicus*. Currently, identification and analysis of the *PAL* family genes have been carried out in multiple plants [89,90,91,92]. In the present study, five members of the PAL family with similar structure and same subcellular localization were identified in *A. mongolicus.* In addition, evolutionary analysis revealed that the relationship between *AmPAL* members and *GmPAL* members was the closest, as both *A. mongolicus* and *G*. *max* belong to the leguminous family. Taken together, members of the PAL family of *A. mongolicus* were relatively conservative in gene evolution and structure. Cis-acting elements analysis suggested that the *AmPAL* genes were involved in abiotic stress response and plant hormone response. Although the expression patterns of the five *PAL* genes are different under different stressors, most of them had experienced upregulation. This meant that, under various environmental stress conditions, biosynthesis of PAL and its related phenylpropane metabolism was activated in *A. mongolicus*. These results deepen our understanding of the relationship between the involvement of PAL members in phenylpropane metabolism and plant abiotic stress resistance.

## 5. Conclusions

*A. mongolicus* is a desert shrub with high tolerance of low temperature stress. The present time–course transcriptomic analysis of low temperature stress showed that *A. mongolicus* responded to low temperature stress by altering the transcription of a large number of genes, and then regulating the synthesis and signal transduction pathways of multiple plant hormones, affecting secondary metabolite pathways, especially the biosynthesis of flavonoids. Furthermore, by identifying the family members of the key flavonoid metabolism *PAL* genes and analyzing their structure, evolution, and expression profiles under multiple stressful conditions, the important role of *PAL* genes in the response of *A. mongolicus* to low temperature stress was highlighted. The present study offers substantial references for understanding the mechanisms of the tolerance to low temperatures in *A. mongolicus*.

## Figures and Tables

**Table 1:** Detailed information of the PAL gene family identified in *A. mongolicus*.

Gene Name	Gene ID	Location	Protein Length (aa ^1^)	MW ^2^ (kDa)	pI ^3^	GRAVY ^4^	Instability Index	Subcellular Localization
AmPAL1	EVM0027535.1_R0	Chr1: 753045-755843	718	78.83	5.85	−0.073	35.82/Stable	Cytoplasm
AmPAL2	EVM0018074.1_R0	Chr7: 67714562-67719380	522	56.32	6.77	−0.080	34.52/Stable	Cytoplasm
AmPAL3	EVM0033527.1_R0	Chr8: 79267527-79270643	713	78.24	6.24	−0.141	33.36/Stable	Cytoplasm
AmPAL4	EVM0004500.1_R0	Chr9: 59574348-59578951	724	78.81	6.03	−0.160	35.32/Stable	Cytoplasm
AmPAL5	EVM0014044.1_R0	Chr9: 77877452-77885295	715	77.85	5.97	−0.165	38.76/Stable	Cytoplasm

aa ^1^: number of amino acids; MW ^2^: molecular weight; PI ^3^: isoelectric point; GRAVY ^4^: grand average of hydropathicity.

**Table 2:** The secondary structure prediction of PAL proteins in *A. mongolicus*.

Proteins	α-Helix (%)	β-Turn (%)	Extended Strand (%)	Random Coil (%)
AmPAL1	54.46%	5.15%	5.29%	35.10%
AmPAL2	47.51%	0.00%	6.90%	45.59%
AmPAL3	55.12%	0.00%	5.19%	39.69%
AmPAL4	52.62%	0.00%	4.97%	42.40%
AmPAL5	52.73%	0.00%	5.17%	42.10%

## Data Availability

The transcriptome data in this study were deposited in GenBank under 15 consecutive accession numbers: SRR24363806, SRR24363807, SRR24363808, SRR24363809, SRR24363810, SRR24363811, SRR24363812, SRR24363813, SRR24363814, SRR24363815, SRR24363816, SRR24363817, SRR24363818, SRR24363819, SRR24363820. The transcriptome data about different seasons were deposited in GenBank under nine consecutive accession numbers: SRR16479821, SRR16479822, SRR16479823, SRR16479824, SRR16479825, SRR16479826, SRR16479827, SRR16479828, and SRR16479829.

## Supplementary Materials

The following supporting information can be downloaded at: https://www.mdpi.com/article/10.3390/genes15111465/s1, Figure S1: (A) Venn diagram analysis of GO terms of DEGs; (B) Venn diagram analysis of KEGG pathways of DEGs; Figure S2: GO and KEGG enrichment analysis of genes in the lightyellow and lightcyan modules. (A) GO enrichment analysis of lightyellow module genes. The *y*-axis represents the number of genes, and the *x*-axis represents the GO term. The red, green, and blue columns represent biological processes, cellular components, and molecular functions, respectively. (B) KEGG enrichment analysis of the genes in lightyellow module. The *y*-axis represents enriched KEGG pathways, and the *x*-axis represents rich factor. (C) GO enrichment analysis of lightcyan module. (D) KEGG enrichment analysis of lightcyan module; Figure S3: GO enrichment analysis of genes in cluster 19 in trend analysis. (A) Biological Process; (B) Cellular Component; (C) Molecular Function. The *x*-axis represents the gene percent, and the *y*-axis represents the GO term. The number after the bar represents the number of enriched genes, and the number in parentheses represents the *p*-value; Figure S4: GO and KEGG enrichment analysis of genes in cluster 0 in trend analysis. (A) Biological Process; (B) Cellular Component; (C) Molecular Function; (D) KEGG enrichment analysis. The *x*-axis represents the gene percent, and the *y*-axis represents the GO term or KEGG pathway. The number after the bar represents the number of enriched genes, and the number in parentheses represents the *p*-value; Table S1: Primers for qRT-PCR of *AmPAL genes*; Table S2: List of PAL members from *Arabidopsis thaliana*, *Glycine max*, *Oryza sativa*, and *Zea mays*.
