# Supplementary material for: Transcriptome Analysis Reveals the Crucial Role of Phenylalanine Ammonia-Lyase in Low Temperature Response in Ammopiptanthus mongolicus
Source: Genes (Basel). 2024 Nov 13;15(11):1465. doi: 10.3390/genes15111465 (PMC11593641; doi:10.3390/genes15111465)
Supplement: Supplementary file 1 [file genes-15-01465-s001.zip › genes-3295055-supplementary.pdf]

genes MDPI

Supplementary files for

Transcriptome Analysis Reveals the Crucial Role of Phenylalanine Ammonia-Lyase in Low Temperature Response in *Ammopiptanthus mongolicus*

Ning Wang, Yilin Zhu, Yijun Zhou, Fei Gao*, and Suxia Cui *

Table S1. Primers for qRT-PCR of *AmPAL genes*

Primer name	Sequence (5 '- 3')
AmPAL1-F	TGCTGACCGGAGAAAAGACT
AmPAL1-R	AAGCCACATACCCTTCCGTT
AmPAL4-F	TGAGCAACACAACCAGGATG
AmPAL4-R	TCTCCTCCAAATGCCTCAGG
AmPAL5-F	TGTGAGTCAAGTGGCCAAGA
AmPAL5-R	GCACTGCAAGGGTCATCAAT
AmeIF1-F	CTGACATGCGCCGTAGGAACG
AmeIF1-R	CCCTGCTTATGCCAGTCTTTT

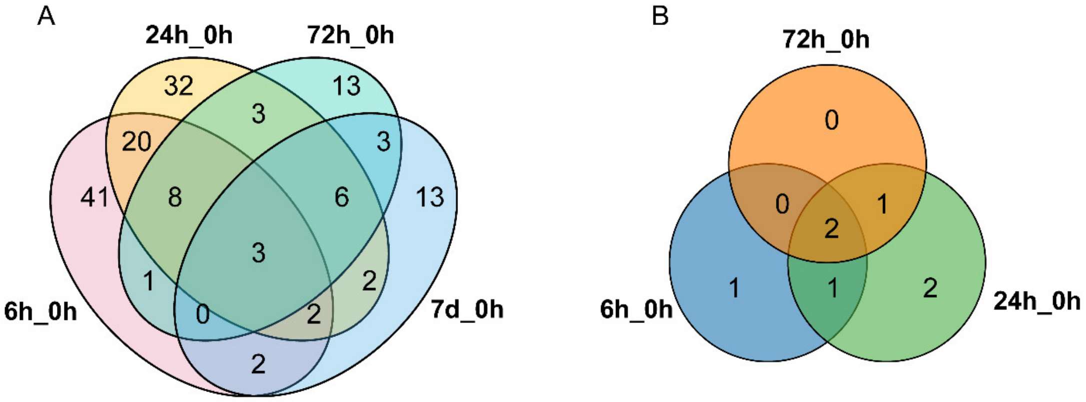

Figure S1. (A) Venn diagram analysis of GO terms of DEGs; (B) Venn diagram analysis of KEGG pathways of DEGs.

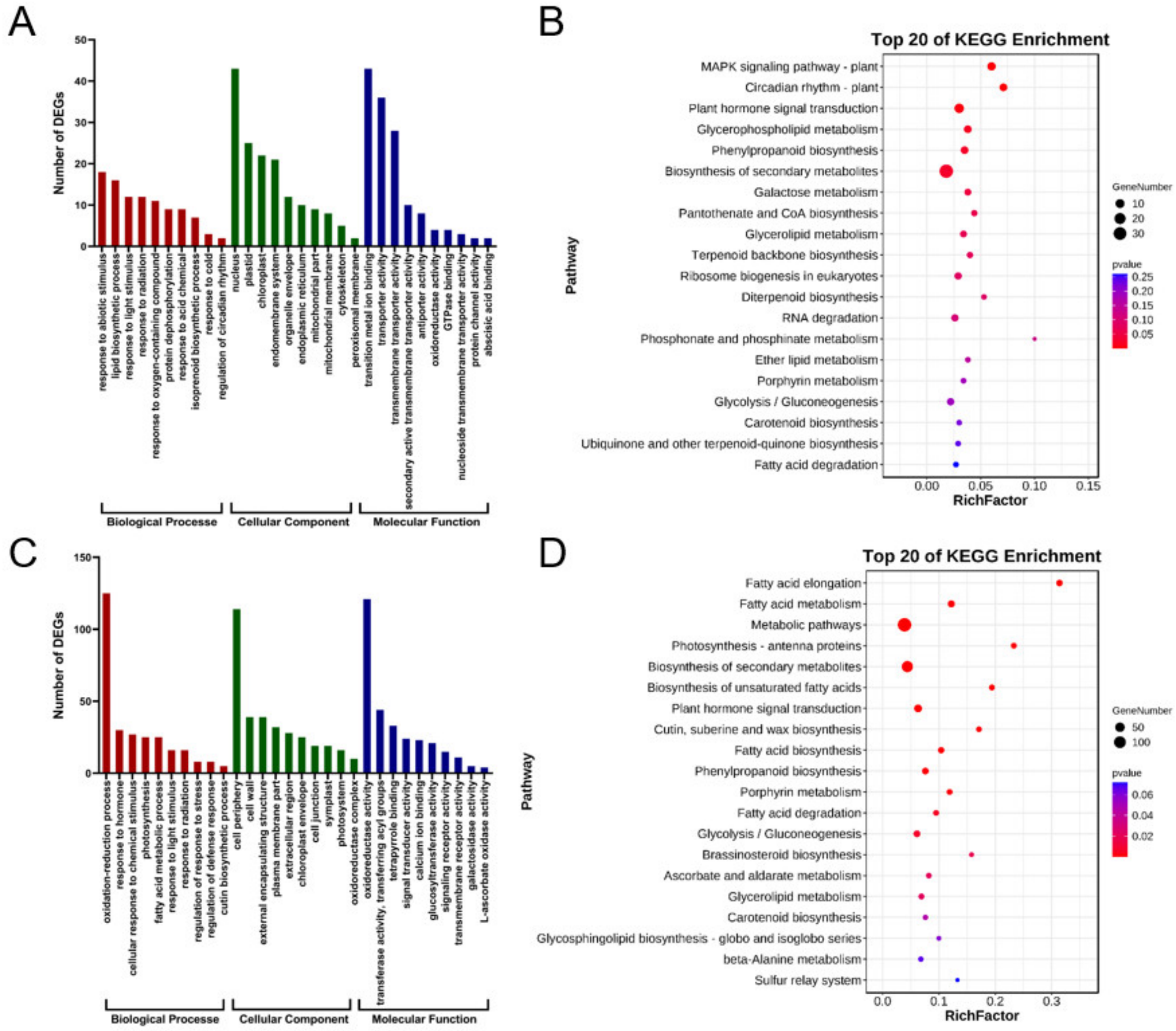

Figure S2. GO and KEGG enrichment analysis of genes in the lightyellow and lightcyan modules. (A) GO enrichment analysis of lightyellow module genes. The y-axis represents the number of genes, and the x-axis represents the GO term. The red, green, and blue columns represent biological processes, cellular components, and molecular functions, respectively. (B) KEGG enrichment analysis of the genes in lightyellow module. The y-axis represents enriched KEGG pathways, and the x-axis represents rich factor. (C) GO enrichment analysis of lightcyan module. (D) KEGG enrichment analysis of lightcyan module.

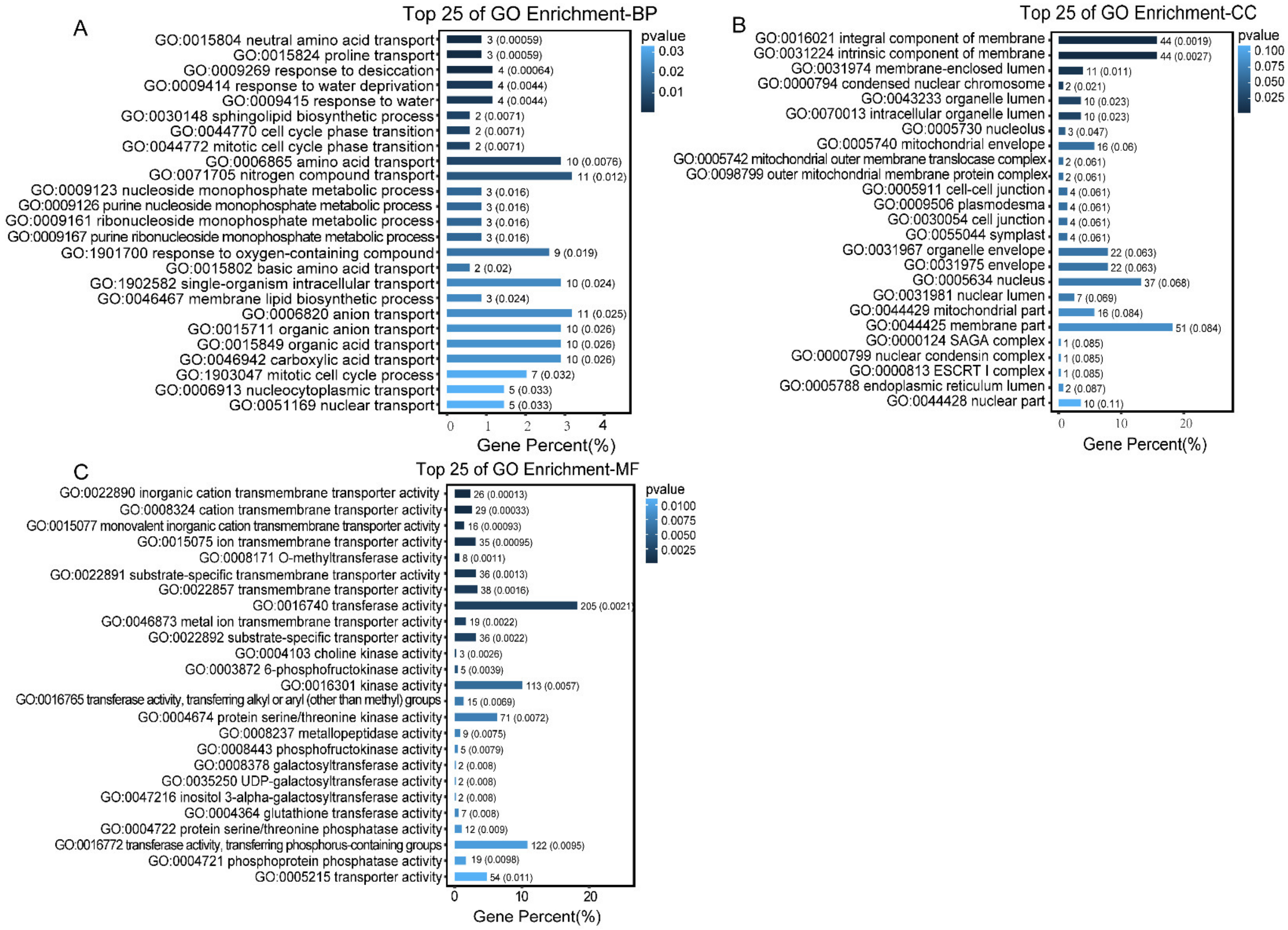

Figure S3. GO enrichment analysis of genes in cluser 19 in trend analysis. (A) Biological Process; (B) Cellular Component; (C) Molecular Function. The x-axis represents the gene percent, and the y-axis represents the GO term. The number after the bar represents the number of enriched genes, and the number in parentheses represents the p-value.

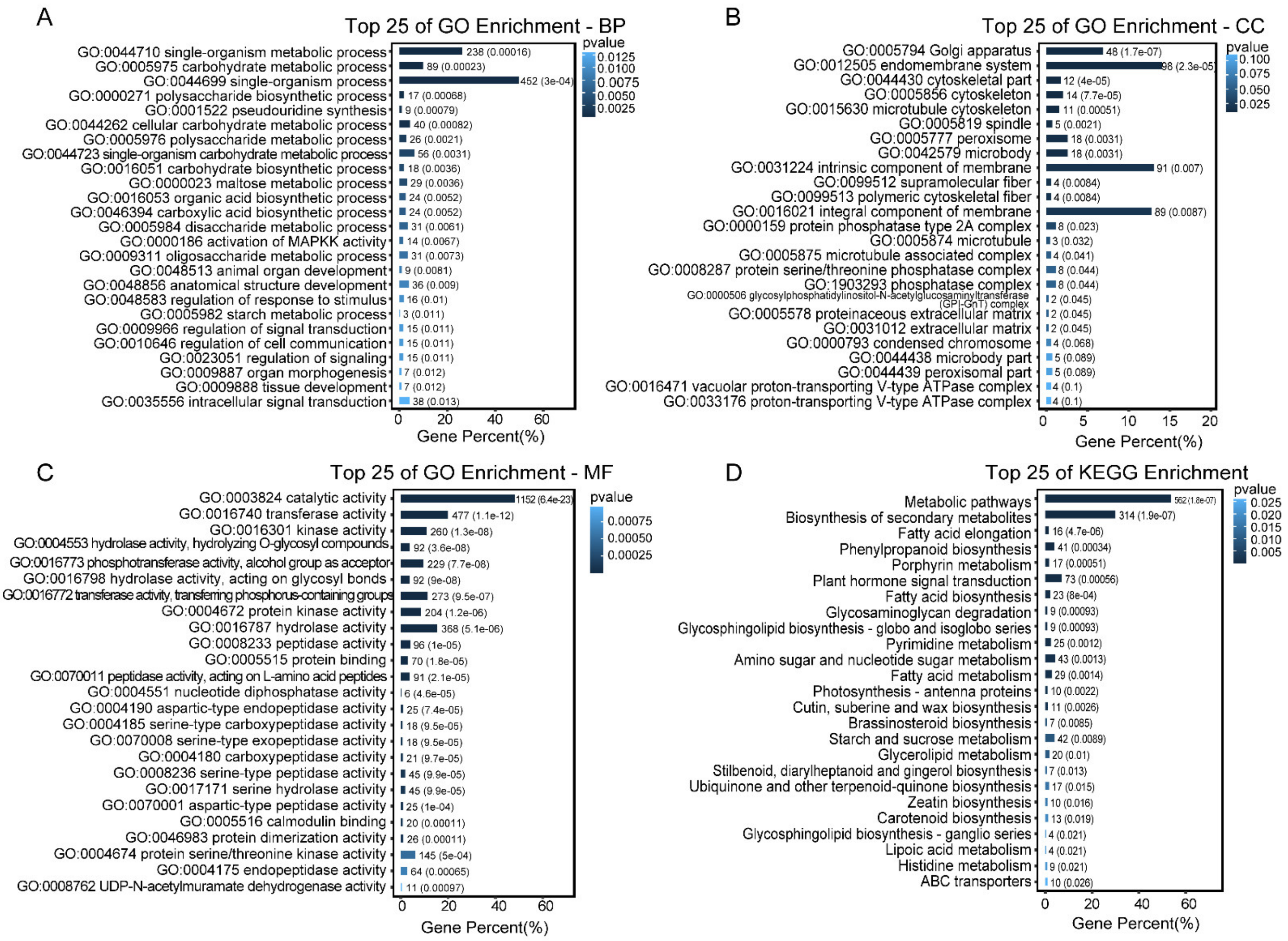

Figure S4. GO and KEGG enrichment analysis of genes in cluster 0 in trend analysis. (A) Biological Process; (B) Cellular Component; (C) Molecular Function; (D) KEGG enrichment analysis. The x-axis represents the gene percent, and the y-axis represents the GO term or KEGG pathway. The number after the bar represents the number of enriched genes, and the number in parentheses represents the p-value.

Table S2. List of PAL members from *Arabidopsis thaliana, Glycine max, Oryza sativa,* and *Zea mays*

Species	Gene name	Gene ID
Arabidopsis thaliana	*AtPAL1*	*AT2G37040*
Arabidopsis thaliana	*AtPAL2*	*AT3G53260*
Arabidopsis thaliana	*AtPAL3*	*AT5G04230*
Arabidopsis thaliana	*AtPAL4*	*AT3G10340*
Glycine max	*GmPAL1.1*	*Glyma.19G182300*
Glycine max	*GmPAL1.2*	*Glyma.03G181700*
Glycine max	*GmPAL1.3*	*Glyma.03G181600*
Glycine max	*GmPAL2.1*	*Glyma.10G058200*
Glycine max	*GmPAL2.2*	*Glyma.20G180800*
Glycine max	*GmPAL2.3*	*Glyma.13G145000*
Glycine max	*GmPAL2.4*	*Glyma.10G209800*
Glycine max	*GmPAL3.1*	*Glyma.02G309300*
Oryza sativa	*OsPAL1*	*LOC_Os02g41630*
Oryza sativa	*OsPAL2*	*LOC_Os02g41650*
Oryza sativa	*OsPAL3*	*LOC_Os02g41670*
Oryza sativa	*OsPAL4*	*LOC_Os02g41680*
Oryza sativa	*OsPAL5*	*LOC_Os04g43760*
Oryza sativa	*OsPAL6*	*LOC_Os04g43800*
Oryza sativa	*OsPAL7*	*LOC_Os05g35290*
Oryza sativa	*OsPAL8*	*LOC_Os11g48110*
Oryza sativa	*OsPAL9*	*LOC_Os12g33610*
Zea mays	*ZmPAL1*	*GRMZM2G074604/Zm00001d017274*
Zea mays	*ZmPAL2*	*GRMZM2G441347/Zm00001d003016*
Zea mays	*ZmPAL3*	*GRMZM2G160541/Zm00001d051161*
Zea mays	*ZmPAL4*	*GRMZM2G063917/Zm00001d051166*
Zea mays	*ZmPAL5*	*GRMZM2G081582/Zm00001d051163*
Zea mays	*ZmPAL6*	*GRMZM2G118345/Zm00001d003015*
Zea mays	*ZmPAL7*	*GRMZM2G170692/Zm00001d017279*
Zea mays	*ZmPAL8*	*GRMZM2G334660/Zm00001d017276*
Zea mays	*ZmPAL9*	*GRMZM2G029048/Zm00001d017275*
